# A Phase 1, Dose-Ranging Study to Assess Safety and Psychoactive Effects of a Vaporized 5-Methoxy-N,N-Dimethyltryptamine Formulation (GH001) in Healthy Volunteers

**DOI:** 10.1192/j.eurpsy.2022.1846

**Published:** 2022-09-01

**Authors:** J. Reckweg, N. Mason, C. Van Leeuwen, S. Tönnes, T. Terwey, J. Ramaekers

**Affiliations:** 1 Maastricht University, Faculty Of Psychology And Neuroscience, Department Of Neuropsychology And Psychopharmacology, Maastricht, Netherlands; 2 Goethe-Universität Frankfurt am Main, Institut Für Rechtsmedizin, Frankfurt am Main, Germany; 3 GH Research PLC, Chief Executive Officer, Dublin, Ireland

**Keywords:** GH001, 5-MeO-DMT, Phase 1, Safety

## Abstract

**Introduction:**

5-Methoxy-N,N-Dimethyltryptamine (5-MeO-DMT) is a tryptamine with ultra-rapid onset and short duration of psychedelic effects. Prospective studies for other tryptamines have suggested beneficial effects on mental health outcomes.

**Objectives:**

In preparation for a study in patients with depression, the present study GH001-HV-101 aimed to assess the impact of four different dose levels of a novel vaporized 5-MeO-DMT formulation (GH001) administered via inhalation as single doses, and in an individualized dose escalation regimen on the safety, tolerability, and the dose-related psychoactive effects in healthy volunteers (n=22). Further, we aimed to assess the impact on cognitive functioning, mood, and well-being.

**Methods:**

The psychedelic experience was assessed with a novel Peak Experience Scale (PES), the Mystical Experience Questionnaire (MEQ), the Ego Dissolution Inventory (EDI), the Challenging Experience Questionnaire (CEQ), and the 5-Dimensional Altered States of Consciousness Questionnaire (5D-ASC).

**Results:**

5-MeO-DMT produced dose-related increments in the intensity of the psychedelic experience ratings on all questionnaires, except the CEQ. Prominent effects were observed following single doses of 6, 12, and 18 mg on PES and MEQ ratings, while maximal effects on PES, MEQ, EDI, and 5D-ASC ratings were observed following individualized dose escalation of 5-MeO-DMT. Measures of cognition, mood, and well-being were not affected. Vital signs at 1 and 3 h after administration were not affected and adverse events were generally mild and resolved spontaneously.

**Conclusions:**

Individualized dose escalation of 5-MeO-DMT may be preferable over single dose administration for clinical applications that aim to enhance the short-term psychoactive effects to elicit a strong therapeutic response.

**Disclosure:**

This study was funded by GH Research PLC, Dublin, Ireland.

